# Physiological insights into ESCRT-mediated phagophore closure: potential cytoprotective roles for ATG8ylated membranes

**DOI:** 10.1080/15548627.2025.2468907

**Published:** 2025-02-24

**Authors:** Kouta Hamamoto, Xinwen Liang, David M. Opozda, Hong-Gang Wang, Yoshinori Takahashi

**Affiliations:** a Department of Pediatrics, Division of Pediatric Hematology and Oncology, The Pennsylvania State University College of Medicine, Hershey, PA, USA; b Department of Pharmacology, The Pennsylvania State University College of Medicine, Hershey, PA, USA

**Keywords:** ATG8ylation, ESCRT, phagophore closure, SQSTM1, TBK1, VPS37A UEVL mutant mouse

## Abstract

The endosomal sorting complex required for transport (ESCRT) machinery is a membrane abscission system that mediates various intracellular membrane remodeling processes, including macroautophagy/autophagy. In our recent study, we established the unique requirement of the ubiquitin E2 variant-like (UEVL) domain of the ESCRT-I subunit VPS37A for phagophore closure, the final step in autophagosome biogenesis, and determined the physiological impact of systemically inhibiting closure by targeting this region in mice. While the mutant mice exhibited phenotypes similar to those reported in mice deficient in generating ATG8 (mammalian Atg8 homologs)-conjugated (ATG8ylated) phagophores, certain phenotypes, such as neonatal lethality and liver injury, were found to be notably milder. Further investigation revealed that ATG8ylated phagophores promote TBK1-dependent SQSTM1 phosphorylation and droplet formation, leading to the formation of large insoluble aggregates upon closure inhibition. These findings suggest potential roles for ATG8ylated membranes in mitigating proteotoxicity by efficiently concentrating and sequestering soluble, reactive microaggregates and converting them into less reactive, insoluble large aggregates. The study highlights VPS37A UEVL mutant mice as a model for investigating the physiological and pathological roles of phagophores that extend beyond degradation.

Macroautophagy (hereafter referred to as autophagy) involves the formation of cup-shaped phagophores, which expand and close to sequester cargo proteins and organelles within double-membrane vesicles known as autophagosomes. One of the most well-characterized features during this process is the conjugation of ATG8-family proteins to the membranes (ATG8ylation), which is critical for the formation and expansion of phagophores. The physiological roles of autophagy have been extensively studied using mice defective in the biogenesis of ATG8ylated phagophores. ATG8ylated membranes also mediate the recruitment and isolation of cytotoxic cargo (e.g., misfolded proteins, malfunctioning organelles) as well as various signaling molecules. Therefore, it is conceivable that blocking autophagy at later stages of autophagosome biogenesis could result in outcomes distinct from those observed when ATG8ylated phagophore formation is inhibited. However, this remains elusive due to limited knowledge of the underlying mechanisms. In our recent study, we established the unique requirement of the ubiquitin E2 variant-like (UEVL) domain of the endosomal sorting complex required for transport (ESCRT)-I subunit VPS37A in phagophore closure and explored the physiological impact of systemically inhibiting autophagy at the final step of autophagosome biogenesis by targeting this domain in mice [1].

Phagophore closure entails membrane scission to separate the inner and outer membranes of the autophagosome. By developing the HaloTag-LC3 autophagosome completion assay, which distinguishes open (phagophores) and closed autophagosomal membranes, we have previously demonstrated the critical role of the ESCRT machinery in this process. The ESCRT machinery consists of protein subcomplexes that assemble into helical filaments, which constrict membrane necks and ultimately induce membrane severing. Although ESCRT is known to mediate a variety of cellular membrane remodeling processes in addition to autophagy, we have found that phagophore closure uniquely requires the ESCRT-I subunit VPS37A. VPS37A is one of four mammalian VPS37 paralogs, each of which contains a modifier of rudimentary/Mod(r) domain capable of forming a heterotetrameric ESCRT-I complex with TSG101, VPS28, and UBAP1-MVB12-associated (UMA) protein. Among the paralogs, VPS37A possesses a distinctive N-terminal region consisting of a UEVL domain flanked by two unstructured regions, which is indispensable for directing the ESCRT machinery during autophagy but not for other processes, such as the endosomal sorting of growth factor receptors. Beyond its role in phagophore closure, the VPS37A N terminus, particularly the N-terminal unstructured region, also functions at a later stage of autophagy, specifically in maintaining the membrane integrity of amphisomes (endosome-fused autophagosomes). Thus, to more precisely disrupt the phagophore closure function of ESCRT, we first narrowed down the VPS37A N-terminal region required for this process. Our data revealed that deletion of the UEVL domain, but not the N-terminal unstructured region, is sufficient to impair the ability of VPS37A to regulate phagophore closure and subsequent autophagic flux. Importantly, the observed phenotypes are unlikely to result from complications due to aberrant sorting of signaling receptors potentially caused by ESCRT-I inhibition, as both ESCRT-I complex formation and EGFR (epidermal growth factor receptor) degradation remain unaffected by the loss of the VPS37A UEVL domain. Moreover, by generating a UMA protein-binding-defective VPS37A mutant, which impairs the incorporation of the endosome-specific ESCRT-I subunit UBAP1 into the complex, we also demonstrated that disruption of the endosomal sorting function of the VPS37A-containing ESCRT-I complex has a minimal effect on autophagy. Collectively, our data show that targeting the VPS37A UEVL domain minimizes the disturbance of other ESCRT-I functions, allowing for a more refined assessment of the effects of phagophore closure inhibition.

To examine the physiological impact of disrupting phagophore closure, we next generated VPS37A UEVL mutant mice in which wild-type VPS37A was systemically replaced with the VPS37A Δ43–139 mutant, retaining the N-terminal unstructured region but lacking the core structural elements of UEVL. The resultant homozygous VPS37A mutant mice were smaller at birth compared to wild-type littermates but were born at the expected Mendelian ratio and did not exhibit other developmental abnormalities or embryonic lethality observed in mice with knockouts of other ESCRT components required for endosomal sorting. However, approximately half of the mutant neonates died shortly after birth, and all surviving mice developed neurological abnormalities, reaching the humane endpoint within three months. Additionally, the mutant mice exhibited other previously characterized autophagy-defective phenotypes, including growth retardation, upregulation of antioxidant genes in the liver, and various tissue abnormalities. Interestingly, despite the systemic accumulation of the autophagy receptor SQSTM1 and polyubiquitinated proteins, several phenotypes, specifically early postnatal lethality and liver injury, were found to be less severe than those reported in ATG8ylation-defective mice. Because ATG7 loss does not further block the minor bulk autophagic flux detected in embryonic fibroblasts derived from the mutant mice, the observed milder phenotypes are unlikely to be attributed to differences in the inhibitory levels of autophagic degradation. Instead, we found that VPS37A mutant cells accumulate SQSTM1/p62 droplets at much higher levels compared to ATG7-deficient cells, and this accumulation is rather reversed by ATG7 loss. The enhanced accumulation of SQSTM1/p62 droplets in the mutant cells is accompanied by an increase in active, phosphorylated TBK1 (TANK-binding kinase 1) on ATG8ylated phagophores, which we found to mediate SQSTM1/p62 phosphorylation and promote its condensation, leading to the formation of detergent-insoluble inclusions. Given that larger aggregates are less reactive than small soluble microaggregates, our findings suggest that ATG8ylated membranes may play a cytoprotective role by facilitating their formation through the recruitment of TBK1 (Figure 1). Future studies are warranted to determine the mechanisms governing the recruitment and activation of TBK1 upon VPS37A UEVL loss and whether TBK1-dependent inclusion formation alleviates proteotoxicity caused by autophagy deficiency. Because VPS37A was originally identified as a cell growth regulatory protein frequently downregulated in hepatocellular carcinoma, it would also be of interest to investigate whether the proposed role of ATG8ylated phagophores contributes to the progression of VPS37A-deficient tumors.

**Figure 1.**
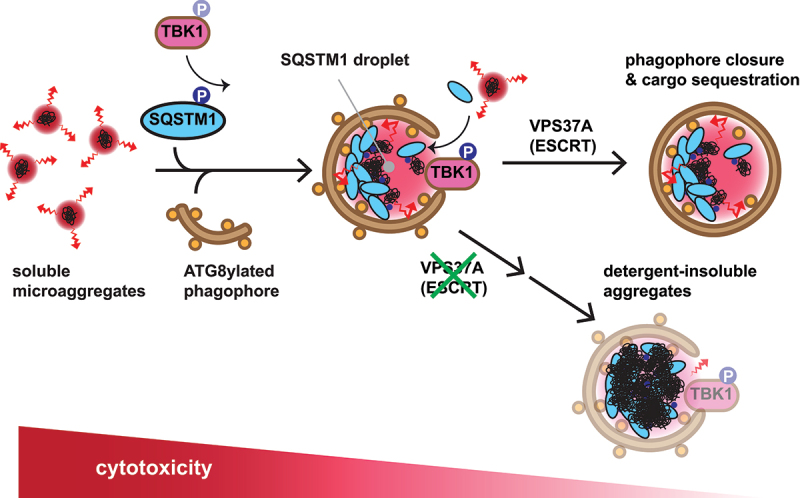
Proposed model for the cytoprotective function of ATG8ylated phagophores. Ubiquitinated misfolded proteins forming reactive, soluble microaggregates are recognized by the autophagy receptor SQSTM1. Phosphorylation of SQSTM1 by TBK1 and other kinases enhances its ubiquitin-binding ability and promotes its condensation into SQSTM1 droplets. ATG8ylated phagophores may play cytoprotective roles not only by enwrapping cytotoxic cargo but also by facilitating further cargo condensation through the phosphorylated, active form of TBK1 on the membranes. As phagophores grow in conjunction with SQSTM1/p62 droplets, the ESCRT machinery seals the membranes and sequesters cytotoxic cargo for lysosomal degradation. The UEVL domain of VPS37A is critical for directing the ESCRT machinery during this process. While impairment of membrane closure prevents complete cargo isolation, the condensed cargo eventually forms less-reactive insoluble aggregates.
